# Comparison of Epithelial Differentiation and Immune Regulatory Properties of Mesenchymal Stromal Cells Derived from Human Lung and Bone Marrow

**DOI:** 10.1371/journal.pone.0035639

**Published:** 2012-05-02

**Authors:** Mario Ricciardi, Giorgio Malpeli, Francesco Bifari, Giulio Bassi, Luciano Pacelli, Armel Hervé Nwabo Kamdje, Marco Chilosi, Mauro Krampera

**Affiliations:** 1 Stem Cell Research Laboratory, Section of Hematology, Department of Medicine, University of Verona, Verona, Italy; 2 Section of Pathological Anatomy, Department of Pathology and Diagnostics, University of Verona, Verona, Italy; University Hospital Freiburg, Germany

## Abstract

Mesenchymal stromal cells (MSCs) reside in many organs including lung, as shown by their isolation from fetal lung tissues, bronchial stromal compartment, bronchial-alveolar lavage and transplanted lung tissues. It is still controversial whether lung MSCs can undergo mesenchymal-to-epithelial-transition (MET) and possess immune regulatory properties. To this aim, we isolated, expanded and characterized MSCs from normal adult human lung (lung-hMSCs) and compared with human bone marrow-derived MSCs (BM-hMSCs). Our results show that lung-MSCs reside at the perivascular level and do not significantly differ from BM-hMSCs in terms of immunophenotype, stemness gene profile, mesodermal differentiation potential and modulation of T, B and NK cells. However, lung-hMSCs express higher basal level of the stemness-related marker nestin and show, following *in vitro* treatment with retinoic acid, higher epithelial cell polarization, which is anyway partial when compared to a control epithelial bronchial cell line. Although these results question the real capability of acquiring epithelial functions by MSCs and the feasibility of MSC-based therapeutic approaches to regenerate damaged lung tissues, the characterization of this lung-hMSC population may be useful to study the involvement of stromal cell compartment in lung diseases in which MET plays a role, such as in chronic obstructive pulmonary disease and idiopathic pulmonary fibrosis.

## Introduction

Mesenchymal stromal cells (MSCs) are multipotent progenitor cells firstly isolated from human bone marrow (BM-hMSCs), displaying differentiation potential into adipocytes, osteoblasts, condrocytes and other tissues of mesodermal origin [1], [2], and specific immune regulatory properties [3], [4]. BM-hMSCs derive from single-cell suspensions by the selective growth of plastic-adherent fibroblast-like cell colonies in liquid culture medium [2]. The easy accessibility of BM-hMSCs, together with their multilineage differentiation potential, raised the hope that these cells could be used for the regeneration of damaged tissues of different origin, including epithelial tissues such as lung. Different reports suggested that BM-hMSCs may differentiate *in vitro* and *in vivo* into epithelial cells [5], [6] through a limited and not fully characterized process called mesenchymal-to-epithelial-transition (MET) [7]–[9]. However, the attempts to induce epithelial phenotype by specific signals, the characterization of the epithelial cells derived from *in vitro* MET and the transplantation of BM-hMSCs *in vivo* into injured lungs led to quite controversial results [10].

MSCs exist in organs other than BM at perivascular level [11], and the tissue origin of MSCs may be responsible for some specific differentiation patterns [12]. The presence of MSCs in human lung has been suggested indirectly by their isolation from fetal lung tissues [13] and bronchioalveolar lavage (BAL) obtained from lung transplanted recipients [14], and by the evidence that the pulmonary stromal compartment possess multilineage differentiation properties [15]. However, lung-derived MSCs (lung-hMSCs) still lack extensive characterization and no data are available about their origin, tissue location, MET capability and immune regulatory properties towards T, B and NK cells.

Aim of this work was to identify, isolate, expand and characterize lung-hMSCs by comparing their features with those already described in BM-hMSCs. In addition, different media containing epithelial differentiation inducers were tested *in vitro* to verify whether lung-hMSCs were more prone than BM-MSCs to acquire specific epithelial tissue-related properties. In particular, retinoic acid (RA) was employed because its involvement in normal lung development [16]; in fact, the disruption of RA signaling leads to major abnormalities in the embryo, including abnormal lung morphogenesis, altered development of the tracheal and bronchopulmonary structures, abnormal differentiation of the respiratory epithelium [17], [18], and lung agenesis [19], [20]. Moreover, it has been recently shown that RA, when administered to adult animals, triggers some genes normally active during lung development, thus ameliorating both functions and structure of damaged lung [21]–[23]. Finally, retinoids are molecular inducers of cell differentiation in many organs and may influence the expression of intermediate filaments such as keratins in different cell types [24]–[26], including MSCs and embryonic stem cells [27], [28]; in addition, RA can reduce the fibrosis occurring after lung injury by down-regulating cytokine secretion and directly inhibiting the proliferation of fibroblasts [29], [30].

## Materials and Methods

### MSC Characterization

BM-hMSCs were obtained from BM aspirates of healthy donors, after written informed consent, as approved by the Ethics Committee of Azienda Ospedaliera Universitaria Integrata Verona (N. Prog. 1828, May 12, 2010 - *‘Institution of cell and tissue collection for biomedical research in Onco-Hematology’*). BM mononuclear cells were separated by density gradient centrifugation and cultured in 25 cm^2^ flasks (BD Biosciences) in 5 ml of culture medium (Dulbecco’s modified Eagle medium (DMEM), 18% fetal bovine serum (FBS), 100 U/ml penicillin and 100 µg/ml streptomycin). Lung-hMSCs were collected from lung explants obtained from living organ donors and not employed for transplantation, according to the Italian legislation concerning organ explantation and under approval of the Ethics Committee of Azienda Ospedaliera Universitaria Integrata Verona (N. Prog. 1899, December 22, 2010 - *‘Institution of pulmonary tissue collection for biomedical research in Oncology and regenerative medicine’*). Human lung tissues were shipped to the laboratory on ice within 12 hours from explantation and then immediately fractionated, enzymatically digested with collagenase and dissociated using gentleMACS™ Dissociator (Miltenyi). At the end of the procedure the single-cell suspension was filtered with a cell strainer, and mononuclear cells were collected by centrifugation and cultured in 25 cm^2^ flasks (BD Biosciences) in the same culture medium used for BM-hMSC expansion. BM- and lung-hMSC cultures were incubated at 37°C in a 5% CO_2_ atmosphere. After 72 hours, non-adherent cells were removed. When 70–80% confluent, adherent cells were washed with phosphate-buffered saline (PBS), trypsinized, harvested, and expanded in larger flasks.

Immunophenotyping of hMSCs was carried out by using monoclonal antibodies (mAbs) specific for CD105, CD106, CD73, CD44 and CD90. In addition, the lack of hematopoietic and endothelial markers (CD31, CD34, CD14, CD45) was assessed. Further characterization of hMSCs was obtained with monoclonal antibodies specific for CD80, CD86, HLA-class I and II, CD117, NG2, Nestin, CD146. All antibodies were purchased from Pharmingen/Becton Dickinson (Milan, Italy). For immunophenotypic analysis, cultured cells were detached by using trypsin/ethylendiaminetetraacetic acid (EDTA), washed with PBS and resuspended at 10^6^ cells/ml. Cell suspension was incubated with specific antibody at room temperature (RT) for 15 minutes. Additional steps with Cytofix/Cytoperm (BD Biosciences) were performed if required, in accordance with supplier’s instructions. Unconjugated mouse IgG1-PE, IgG2-PE and IgG1 + Goat anti-Mouse-PE (all BD Biosciences) were used as relative control isotypes. At least 10,000 events were analyzed by flow cytometry (FACScalibur; Becton Dickinson, Milan, Italy) and Cell Quest Software.

MSC differentiation potential was assessed by testing their ability to differentiate into adipocytes, osteoblasts and chondrocytes in presence of specific differentiation media [31]. Adipocytic differentiation was achieved after 3 week-culture of MSCs with adipogenic medium (AM), containing 10^−6^ M dexamethasone, 10 µg/ml insulin and 100 µg/ml 3-isobutyl-1-Methylxantine. Osteoblastic differentiation was achieved after 2 week-culture with osteoblastic medium (OM) containing 10^−7^ M dexamethasone, 50 µg/ml ascorbic acid and 10 mM µ-glycerophosphate. Chondrocytic differentiation was achieved after 2 week-culture with chondrocytic medium (CM), containing 10^−7^ M dexamethasone and 10 ng/ml TGF-β (SIGMA), which was added to a pellet of 2.5×10^5^ MSCs centrifuged at 1500 rpm for 10 minutes. Oil-red-O, von Kossa and toluidine blue dyes were employed to identify adipocytes, osteoblasts and chondrocytes, respectively.

To assess population doubling time, Lung-hMSCs were plated at 5×10^4^ concentration in duplicate in DMEM at different passages (P3, P5 and P7). Cells were detached and counted after 24, 48 and 72 hours. Data were analyzed by using the formula described at http://www.doubling-time.com/compute.php website to generate exponential regression based on Least Squares Fitting-Exponential.

To perform colony forming unit assay-fibroblast (CFU-F) and self-renewal assay, hMSCs were plated at 4, 20 and 40 cells/cm^2^ concentrations. After 14 days of culture, every single cell-derived cluster with more than 50 cells was considered as colony. CFU-F assay was performed at passage P2, P3, P4, P5 and P6. CFU-F colonies were stained with May-Grunwald Giemsa and then counted. Self-renewal assay was performed by replating primary colonies and verifying that these cells were capable of forming secondary colonies.

For immune regulation assays, human peripheral blood mononuclear cells were obtained from healthy blood donors after written informed consent, as approved by the Ethics Committee of Azienda Ospedaliera Universitaria Integrata Verona (N. Prog. 1828, May 12, 2010 - *‘Institution of cell and tissue collection for biomedical research in Onco-Hematology’*); human B, NK and T cells were selected by using negative immunomagnetic selection kits (Miltenyi Biotec, Calderara di Reno (BO), Italy). Cell purity was evaluated by using specific monoclonal antibodies and flow cytometry; only samples with purity >95% were used. Proliferation of stimulated B, NK and T cells in coculture with BM- and lung-hMSCs was evaluated at day 4 (B and NK cells) and at day 7 (T cells) by means of CFDA-SE dilution method (Invitrogen). All effectors were labelled with CFDA-SE [5 µM] and then cocultured with hMSCs. At the end of the coculture, lymphocytes were stained with anti-CD45 mAb (Beckton Dickinson, Milan Italy) and Topro3 (Invitrogen) and analyzed. B cells were cocultured in RPMI + 10% FBS (Gibco) and stimulated with CD40 ligand and its enhancer MAB050 (R&D System), IL-2 (Proleukin, Chiron Corporation), mouse F(ab) anti-human IgA/M/G (Jackson Immunoresearch) and CpG 2006 (Invivogen). NK cells were cocultured with IMDM + 10% Human Serum and stimulated with IL-2 (Proleukin, Chiron Corporation). T cells were cocultured in RPMI + 10% Human Serum and stimulated by using mitogenic anti-CD3 [0.5 µg/ml] and anti-CD28 [0.5 µg/ml] mAbs (Sanquin, Pelicluster).

In the experiments with inhibitors of MSC immune modulatory pathways [32], the following molecules were added after T cell activation: indoleamine-2,3-dioxygenase-1 (IDO) inhibitor (L-1-methyl-tryptophan, L-1MT - Sigma Aldrich) [1 mM]; inhibitor of heme-oxigenase 1 (snPP - Frontier Scientific) [2 µM]; inhibitor of COX2, an inducible enzyme involved in the production of prostaglandin (PG)-E2 (NS398 - Cayman) [5 µM]; inhibitor of inducible nitric oxide synthase - iNOS (L-NMMA - Cayman) [1 mM]; IFN-gamma blocking antibody (BD Phanrmingen) [10 µg/ml].

To assess epithelial differentiation, hMSCs from bone marrow and lung at passage P4 were cultured for 4 weeks with DMEM low-glucose+5% FBS (Gibco) + 1% Pennicillin/Streptomycin (Gibco) + Retinoic Acid (Sigma) 10 µM. At the end of culture, cells were either used still adherent for in situ immunofluorescence or trypsinized and washed with PBS for the assays on cell suspension. Other methods were tested, such as RA in combination with other factors (EGF, epidermal growth factor; IGF - insulin growth factor; HGF - hepatocyte growth factor; FGF10 - fibroblast growth factor 10), as well as the addition of supernatant from immortalized epithelial cell lines (16hbe human bronchial epithelial cell line; A549 human carcinoma - alveolar basal epithelial cell line).

For quantitative reverse transcription polymerase chain reaction (qRT-PCR), total RNA was isolated from cells with TRIzol Reagent (Invitrogen) and retrotranscribed with High Capacity cDNA Reverse Transcription Kit (Applied Biosystems), according to user’s manuals; qRT-PCR was carried out in 10 µl total volume containing 5 ng of cDNA and 400 nM of each primer in 1x Power SYBR Green I Master Mix (Applied Biosystems). After a starting denaturation for 10 minutes at 95°C, 40 PCR cycles (15s 95°C and 1 min 60°C) were performed by ABI PRISM 7900HT SDS instrument (Applied Biosystems). Oligonucletide primers used in qRT-PCR are listed in Table 1. Each sample was evaluated in triplicate; specificity of PCR products was checked at the end of each run by melting curve analysis. The probe signal was normalized to the internal reference, and a cycle threshold was taken above the background fluorescence. Relative quantification of gene expression was calculated by using *ACTB* level as endogenous reference. Expression data were analyzed by the comparative method, as shown in User Bulletin #2 (Applied Biosystems). Immortalized, human bronchial epithelial cell line (16hbe) was used as internal control for epithelial markers.

**Table 1:** Oligonucleotides used for quantitative RT-PCR (qRT-PCR).

	Oligonucleotide (5′-3′)	PCR product
*NES*, Nestin	F- GCG GTGGCTCCAAGACTTCR- ACTGGGAGCAAAGATCCAAGAC	100 bp
*SMAD4,* SMAD family member 4	F- TTCTGGAGGAGATCGCTTTTGR- TTGCCTATGTGCAACCTTGCT	86 bp
*NANOG,* Nanog homeobox	F- GCAATGGTGTGACGCAGAAGR- AGGTTCCCAGTCGGGTTCA	94 bp
*POU5F1,* POU class 5 homeobox 1	F- ACCCACACTGCAGATCAR- CCACACTCGGACCACATCCT	70 bp
*SOX2,* SRY (sex determining region Y)-box 2	F- CCGTTCATCGACGAGGCTAAR- TCATGAGCGTCTTGGTTTTCC	97 bp
*TDGF1*, teratocarcinoma-derived growth factor 1	F- AGAACCTGCTGCCTGAATGGR- AGTTCTCTTTGCGCACATCGT	106 bp
*OCLN*, occludin	F- CCTATAAATCCACGCCGGTTCR- AACGAGGCTGCCTGAAGTCA	81 bp
*KRT18P19,* cytokeratin 18	F- TGGAGAAGAAGGGACCCCAR- TTGCGAAGATCTGAGACCTC	81 bp
*TJP1*, tight junction protein 1	F- CATCAGATCATTCTGGTCGATCAR- TCCGGAGACTGCCATTGC	132 bp
*VIM*, vimentin	F- TCCAAGTTTGCTGACCTCTCTGR- CAGTGGACTCCTGCTTTGCC	76 bp
*SNAI1*, snail homolog 1 (Drosophila)	F- TGCATATTCGGACCCACACAR- TGTTGCAGGAGGGCAAGAA	132 bp
*ACTB*, actin beta	F- CGCGAGAAGATGACCCAGATR- GTCACCGGAGTCCATCACG	125 bp

Legend: F: forward; R: reverse; bp: base pairs.

For tissue immunofluorescence, fresh lung tissue was washed with sterile PBS, fixed in paraformaldehyde at room temperature for 2 hours and, after three washes with PBS, dehydrated in 30% sucrose overnight at 4°C. After complete de-hydration, the tissue was gradually frozen and embedded in tissue freezing medium (OCT). Sections (8 µm) were cut by a cryostat (Leica CM1850) and stored at −20°C. For immunofluorescent staining, sections were incubated with blocking solution containing 5% FBS (GIBCO) + 0.2% Triton X-100 (Sigma) in PBS for 1 hour at RT. Incubation with uncoupled primary antibodies was carried out overnight at 4°C; the secondary antibodies, diluted in blocking solution, were incubated for 2 hours at RT. After wash in PBS, samples were stained with topro3 (1∶3,000) (Sigma) for 10 minutes and mounted with the anti-bleaching reagent DABCO (Invitrogen). The following uncoupled anti-human primary antibodies were used: polyclonal rabbit anti-human NG2 (Millipore), monoclonal mouse anti-human-Nestin (R&D), monoclonal mouse anti-human CD31 (DAKO), monoclonal mouse anti-human CD146 (Novocastra). Secondary Cy3-labelled goat anti-mouse and 488-donkey anti-rabbit mAbs were also used (Jackson Immunoresearch). Nuclei were stained with Topro3 (Invitrogen, 1∶3,000).

For in situ cell immunofluorescence, hMSCs at P4 were seeded on sterile glass-culture chambers (Nunc). At the end of epithelial differentiation protocol, culture medium was removed and cells were fixed in PBS + 4% paraformaldehyde + 4% sucrose. Samples were washed in PBS, incubated 30 minutes in blocking solution (PBS + BSA 1% + Triton X-100 0.2%) and then 90 minutes with primary antibody in blocking solution at RT. After 3 washes in blocking solution, samples were incubated 60 minutes with secondary antibody in blocking solution at RT. If required, samples were treated with Zenon Mouse IgG1 Labeling Kits (Invitrogen) to reveal secondary mouse anti-human mAbs in double immunofluorescence, in accordance with Supplier’s instructions. Cells and lung sections were washed in PBS, stained with topro3 (1∶3,000) (Sigma) for 10 minutes and mounted with the anti-bleaching DABCO reagent (Invitrogen). The following antibodies were used:

mouse anti-human Cytokeratin 18 (Pierce), mouse anti-human E-cadherin (Dako), mouse anti-human Smooth Muscle Actin (Dako; M0851), and mouse anti-human Occludin (Santa Cruz; sc-81812) mAbs.
Goat anti-mouse Alexa Fluor 546-conjugated secondary antibody (Molecular Probes A11032, batch 419360, 1∶1,000).

Images were acquired with Zeiss LSM 510 confocal microscope equipped with argon (488 nm) and helium/neon (543 nm) excitation lasers. For 488 nm excitation, emission was selected at 510–530 nm bandpass filter, whereas for 546 nm excitation emission was selected by using a 560 nm longpass filter.

For polyacrylamide gel electrophoresis and western blotting, cellular proteins were harvested by using deoxycholate (DOC) and quantified by using a BCA protein assay (Pierce). Equal amounts of proteins were loaded on 10% polyacrylamide gel and transferred to a nitrocellulose membrane. After blocking with 3% milk in Tris-Buffered Saline + Tween 0.1% (TBS-T), membranes were incubated with primary antibodies overnight and stained with anti-mouse IgG horseradish peroxidase-conjugated antibodies (Sigma) for 1 hour. Protein levels were detected by chemio-luminescence (Pierce). Monoclonal mouse anti-human Cytokeratin 18 (Pierce) and monoclonal mouse anti-human Vimentin (Dako) mAbs were used as primary antibodies. Beta-actin was used as marker of protein loading (monoclonal mouse anti-human beta actin - Sigma).

Tight junction integrity in BM-hMSCs and lung-hMSCs was assessed during epithelial differentiation by measuring the transepithelial electrical resistance (TEER) with an EVOM epithelial voltmeter (World Precision Instruments, Sarasota, FL, USA). 5×10^5^ cells were seeded on Transwell® filter inserts (Cell Culture Insert 0.4 µm, Falcon) in 24 multi-well plates (Falcon). TEER measurement on hMSCs was performed 3 times a week during the 4 weeks of epithelial differentiation. The apical volume in the Transwell® system was 200 µl and the basal volume in the multi-well was 700 µl. Data were normalized in Ω×cm^2^. The background of the filter inserts (45±5 Ω×cm^2^) was subtracted from other measures.

### Statistical Analysis

Data were expressed as mean ± standard deviation (SD). Differences among the experimental conditions were analyzed by using non-parametrical tests (Wilcoxon and Kruskal-Wallis with Dunns post test for multiple comparisons). P value <0.05 was considered statistically significant.

## Results

### Isolation and Characterization of Lung-hMSCs

**Figure 1:**
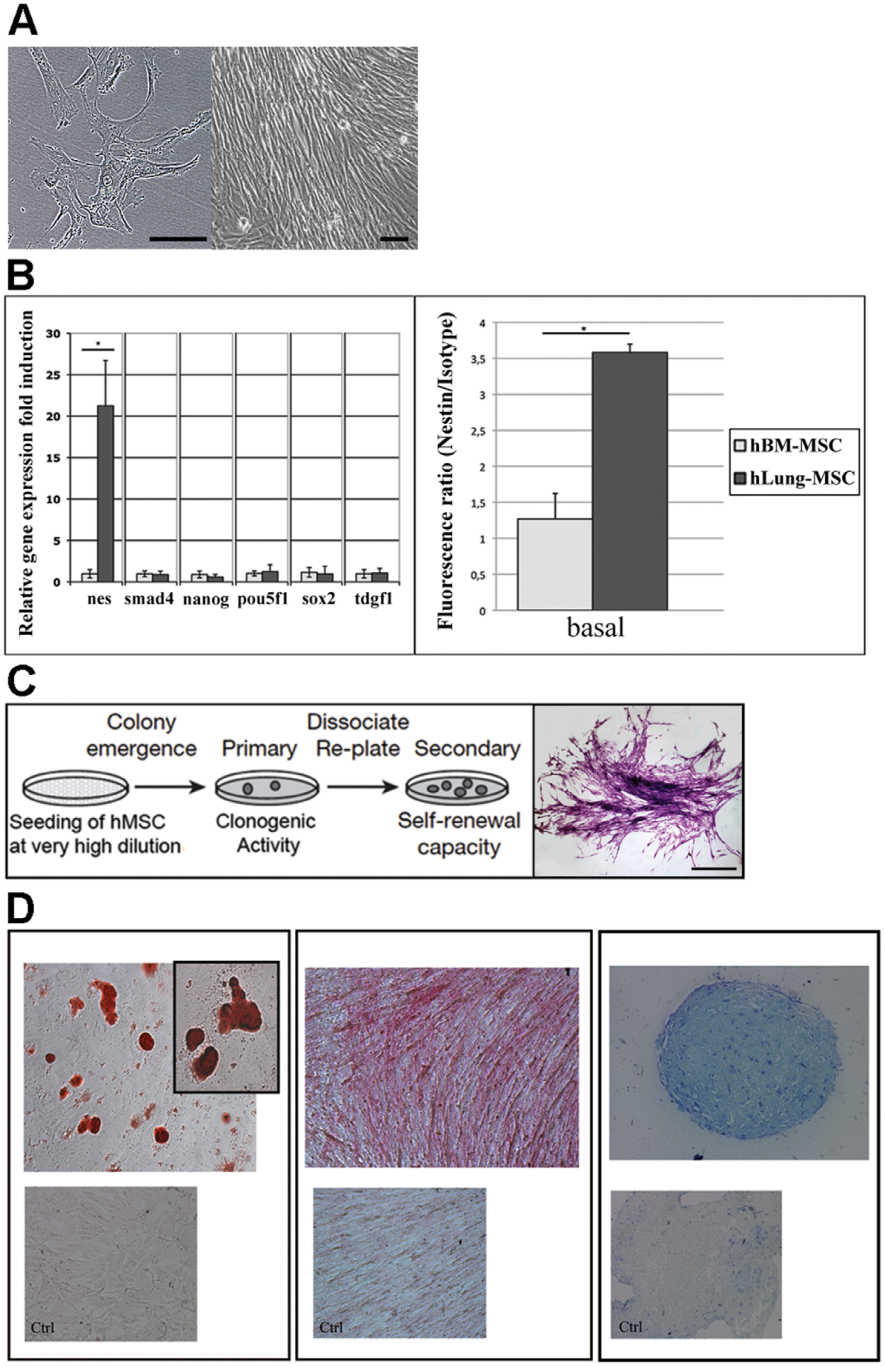
Characterization of lung-hMSCs. (**A**) Morphology of lung-hMSCs. Inverted phase-contrast microscopy of primary colonies of lung-hMSCs (*left panel*) and expanded colonies at confluence (*right panel*). (**B**) (*left panel*) Characterization by qRT-PCR of stemness profile of cells extracted from lung tissues (dark grey columns) in comparison with BM-hMSCs (ligh grey columns). Relative gene expression fold induction of nestin (nes), smad4, nanog, pou5f1(or Oct4), sox2 and tdgf1 (or Cripto). Gene expression is normalized on actb gene (beta actin) and related to BM-hMSCs as basal expression. Mean ± SD; * = p<0.01; (**B**) (*right panel*) Analysis of nestin expression detected by flow cytometry in lung-hMSCs (dark grey column) and BM-hMSCs (light grey column). Columns show Nestin fluorescence ratio normalized on the fluorescence of the relative isotype. Mean ± SD. * p<0.01. (**C**) Schematic representation of clonogenic and self-renewal assays with a representative case of a single colony obtained from CFU-F assay, consisting of more than 50 cells. Scale bar: 1 mm. (**D**) Multilineage differentiation potential. Lung-hMSCs, under appropriate stimuli, can generate adipocytes (*left panel*), osteocytes (*middle panel*) and chondrocytes (*right panel*), as confirmed by the staining with Oil-red-O, alkaline phosphatase (ALP) and Tolouidin Blue dyes, respectively.

Cell suspensions obtained after disaggregation of fresh human lung samples and plated in culture medium gave rise after 7–10 days to multiple colonies of plastic-adherent fibroblast-like cell colonies with the same morphological features of CFU-F obtained with BM-hMSCs (Figure 1A). Adherent cells were enzymatically disaggregated and re-plated; within 2–5 passages from the initial plating of the primary culture, lung-derived adherent cells displayed the typical spindle-shaped, fibroblastic morphology of BM-MSCs (Figure 1A). These cells showed high proliferative rate, with an average population doubling time of 34.6±13.0 hours (data not shown).

Immunophenotyping for surface antigens was performed by flow cytometry on lung-derived adherent cells and BM-hMSCs at passages 2–7 (Table 2). Lung-derived cells, as well as BM-hMSCs, were negative for hematopoietic, endothelial and monocyte markers (CD45, CD31, CD34, CD117, CD14) and costimulatory molecules (CD80, CD86). In addition, both cell types expressed markers of mesenchymal progenitors (CD44, CD73, CD90, CD105 and CD106) and perivascular cells (CD146 and NG2), and resulted positive for HLA class I (Table 2).

**Table 2:** Characterization of BM-hMSCs and lung-hMSCs by flow cytometry. Data are expressed as marker/isotype fluorescence ratio (n = 6).

	BM-hMSC	lung-hMSC
**CD45**	1±0.03	1±0.08
**CD31**	1±0.1	1±0.03
**CD34**	1±0.03	1±0.07
**CD14**	1±0.08	1±0.03
**CD80**	1±0.1	1±0.2
**CD86**	1±0.06	1±0.08
**CD44**	173±82	141±3.8
**CD73**	44±27	55±12
**CD90**	396±111	386±178
**CD105**	7±5.1	3,8±1.2
**CD106**	81±5.7	1.6±0.1
**CD117**	1±0.04	1±0.06
**CD146**	16±8	6.5±4.2
**NG2**	10±1	11±1.6
**HLA-I**	41±3	24±1.2
**HLA-II**	1±0.1	1±0.2

The expression at mRNA level of a panel of stemness-related genes was evaluated by qRT-PCR in lung-derived adherent cells and BM-hMSCs (Figure 1B). The expression of SMAD4, NANOG, POU5F1 (also named oct4), SOX2 and TDGF1 (also named cripto) was not significantly different in the two cell populations; by contrast, NES (Nestin-coding gene) expression resulted 21 times-higher in lung-derived adherent cells (P<0.01) (Figure 1B). Cytofluorimetric analysis of Nestin protein, normalized on the relative isotype, confirmed the higher expression of Nestin in the cells obtained from lung tissues (P<0.01) (Figure 1B).

CFU-F assay performed with cells derived from lung tissue and BM-hMSCs led to similar results (19.4±1.2% and 20.1±2.0%, respectively, n = 6). In addition, self-renewal capacity of adherent cells derived from lung tissue was demonstrated by dissociating and replating primary colonies that gave rise to secondary colonies similarly to BM-MSCs (Figure 1C). Finally, the ability to differentiate into multiple mesodermal lineages, i.e. adipocytes, osteocytes and chondrocytes, was similar in the two cell populations (Figure 1D). According to all these results, these cells could be properly named lung-hMSCs.

**Figure 2:**
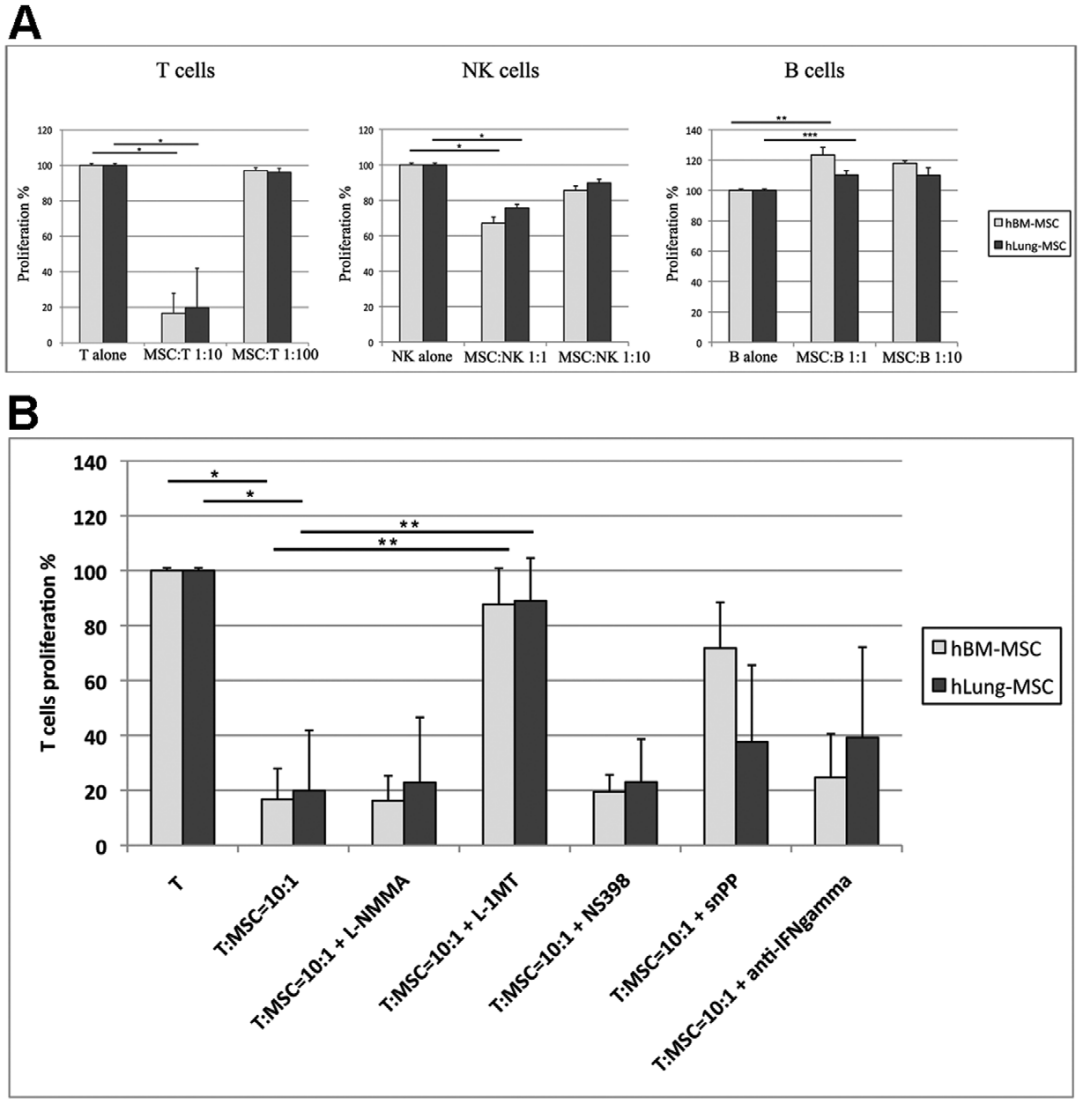
Immune regulatory properties. (**A**) Effect of the coculture of MSCs derived from human BM (light grey) and human lung (dark grey) on the proliferation of B, NK and T cells. Different MSC:Effector ratios were assessed as shown in the legend. Means±SD. * p<0.01, ** p<0.03. (**B**) Molecular mechanisms underlying the immune regulatory properties of BM-hMSCs (light grey) and lung-hMSCs (dark grey) towards T cells. Proliferation of T cells was evaluated without MSCs or at 10∶1 ratio with hMSCs from BM and lung, in presence of specific inhibitors of known pathways: IDO-inhibitor L-1-methyl-tryptophan (L-1MT), inhibitor of heme-oxigenase 1 (snPP), COX2 -inhibitor (NS398), iNos-inhibitor (L-NMMA) and IFN-gamma blocking antibody.

Activated T cells were cocultured with lung-hMSCs for 6 days, as described in Materials and Methods. Similarly to BM-hMSCs, lung-hMSCs displayed their inhibitory effect on T cell proliferation at 1∶10 MSC/T cell ratio, whereas at 1∶100 ratio no effect was observed. The differences in the inhibitory effect between the two hMSC types were not statistically significant (Figure 2A, *left panel*).

Similar inhibitory capacity of lung-hMSCs and BM-MSCs was found by coculture assays with activated NK cells for 5 days: both hMSC types exerted the maximum inhibitory effect on NK cell proliferation at 1∶1 MSC/NK cell ratio, whereas no significant effect was observed at 1∶10 ratio (Figure 2A, *middle panel*).

Coculture of lung-hMSCs with B cells showed that lung-hMSCs and BM-hMSCs have similar supportive effect towards B cells. In fact, B cell proliferation was enhanced by the coculture with either lung-hMSCs or BM-hMSCs. No significant differences were observed in the enhancement of B cell proliferation mediated by both hMSC types (Figure 2A, *right panel*).

The molecular mechanism underlying the immune regulatory effect of hMSCs on T cell proliferation was also investigated by using specific inhibitors [17]. IDO resulted the pivotal mechanism involved in hMSC immunomodulation, as the addition of its specific inhibitor L-1MT completely reverted the modulatory effect (Figure 2B). In addition, heme-oxigenase 1 (inhibited by snPP) and IFN-γ (inhibited by specific blocking antibodies) resulted partially involved in the regulatory effect of hMSCs, although in a non-statistically significant manner; by contrast, no effect was observed by using COX2 inhibitor to block PGE2 production (NS398) and iNOS inhibitor (L-NMMA) (Figure 2B).

**Figure 3:**
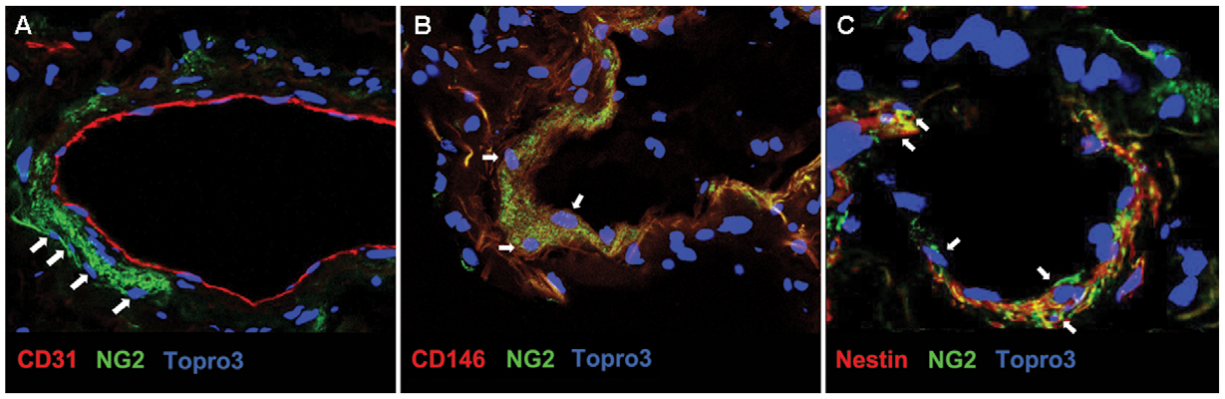
In vivo localization of lung-hMSCs at perivascular level. Immunofluorescence on human lung tissue sample with anti-NG2 and anti-CD31 *(left panel)*, anti-NG2 and anti-CD146 *(middle panel)*, and anti-NG2 and anti-Nestin *(right panel)* mAbs. White arrows: cells of interest. Nuclei are stained with Topro3 in blue.

Immunofluorescence on lung sections was performed by using anti-NG2, -CD146, -CD31 and -Nestin antibodies to identify *in situ* lung-hMSC niches; the co-expression of NG2 and CD146 was used to detect lung-hMSCs. Double-positive cells were found surrounding endothelial cells (CD31-positive cells) at perivascular level; in addition, NG2-positive cells co-expressed Nestin protein (Figure 3), thus confirming the results obtained by qRT-PCR and FACS analysis performed on cultured cells.

### Epithelial Differentiation Potential of Lung-hMSCs

**Figure 4:**
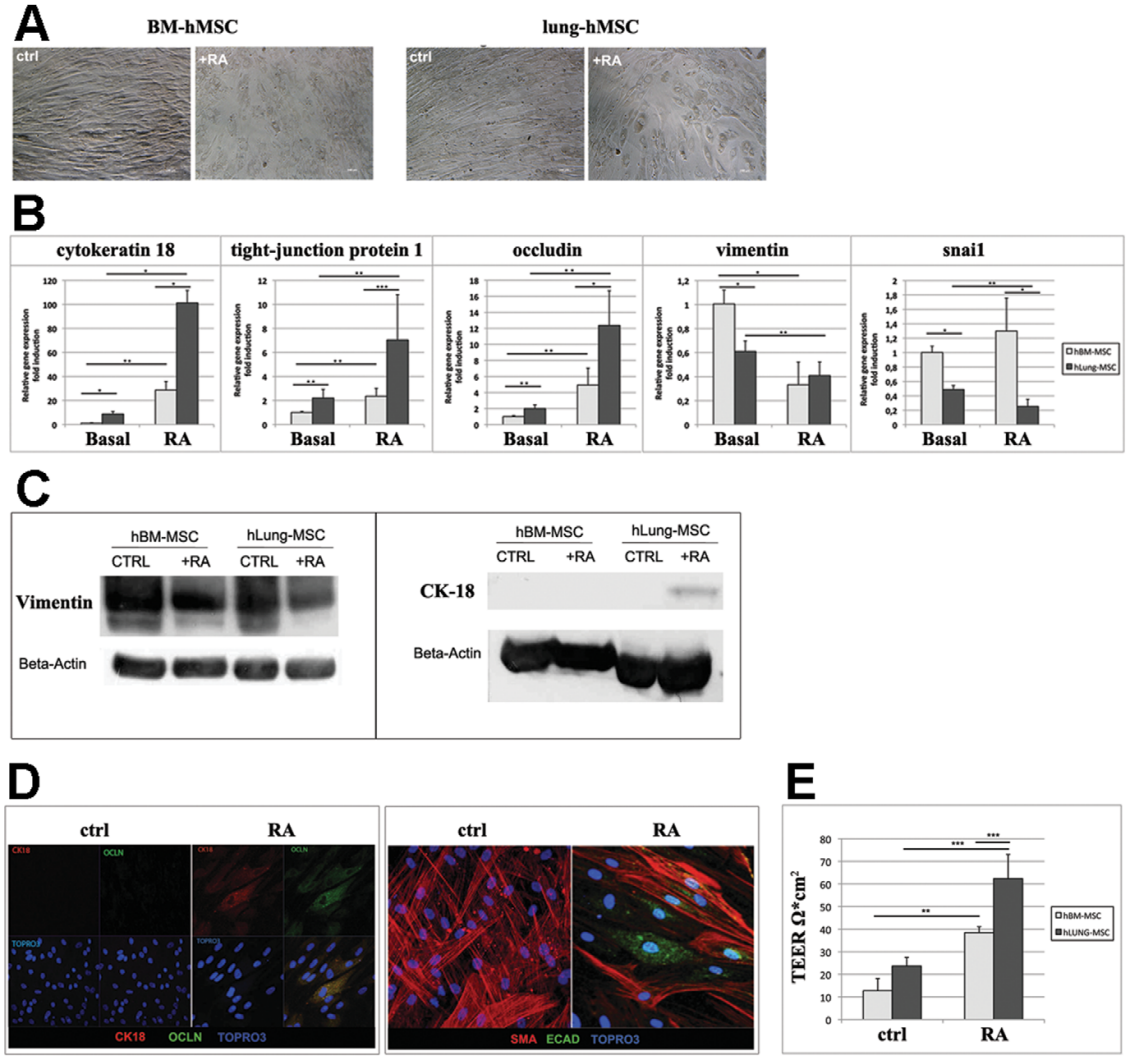
Epithelial differentiation. (**A**) Morphology changes after epithelial differentiation. Inverted phase-contrast microscopy (Zeiss Observer.Z1; 100x) of BM-hMSCs and lung-hMSCs without and with RA treatment (*Ctrl; +RA*). (**B**) Evaluation of epithelial differentiation by qRT-PCR on BM-hMSCs (light grey) and lung-hMSCs (dark grey), before (basal) and after RA treatment for the induction of epithelial differentiation. Panel shows the expression of the epithelial genes cytokeratin 18 (krt18), tight junction protein (tjp1), also named zona occludens 1, occludin (ocln) and mesenchymal genes vimentin (vim) and e-cadherin repressor snai1. Data are normalized on actb (beta actin) expression and related to basal BM-hMSCs. The error bars represent standard deviation; * =  p<0.01; ** = p<0.03; *** = p<0.05. (**C**) Evaluation of epithelial differentiation by Western Blot with anti-vimentin (*left panel*) and anti-cytokeratin 18 (*right panel*) antibodies on protein extract of BM-hMSCs (lane 1-2) and lung-hMSCs (lane 3–4), either untreated (Ctrl) or treated with RA (+RA). (**D**) Evaluation by immunofluorescence of epithelial differentiation of lung-hMSCs after RA treatment. Staining with anti-cytokeratin 18 (CK18) and Occludin (OCLN) (*left panel*), and with E-cadherin (Ecad) and anti-smooth muscle actin (SMA) (*right panel*) mAbs. Nuclei are stained with Topro3 in blue. (**E**) Functional evaluation of epithelial differentiation with Trans-Epithelial Electric Resistance (TEER). Histograms show the value of TEER (Ω*cm^2^) on BM-hMSCs (light grey) and lung-hMSCs (dark grey), before and after RA treatment. The error bars represent standard deviation; ** = p<0.03; *** = p<0.05.

Only modest results could be obtained in absence of RA, i.e. the partial upregulation of the cytokeratin 18 at lower levels than with RA. On the other hand, the addition of FGF10 to RA did not enhance significantly epithelial differentiation. Similarly, supernatant from either 16hbe or a549 immortalized epithelial cell lines induced the upregulation of specific epithelial markers but not organized MET (data not shown). Thus, to assess whether lung-hMSCs possess some tissue-specific properties, we focused our attention on the effects of RA on lung-hMSCs and BM-hMSCs. Both MSC types were cultured for 4 weeks with RA, as described in Materials and Methods, and the effect of RA on hMSCs was analyzed at phase-contrast microscopy. As shown in Figure 4A, both lung-hMSCs and BM-hMSCs acquired at the end of culture a round-cuboid shape resembling a monolayer of epithelial cells, but these features were more remarkable in lung-hMSCs. Cells maintained the typical mesenchymal shape in the controls (untreated MSCs from lung and BM) (Figure 4A). The expression of three epithelial genes (cytokeratin 18, occludin and tight-junction protein) and two mesenchymal genes (vimentin and snai1), known to be involved in the epithelial-mesenchymal transition [7], [8], were evaluated by qRT-PCR in MSCs cultured in presence of RA. Untreated lung-hMSCs showed higher basal expression of the three epithelial transcripts, as compared to untreated BM-hMSCs (Figure 4B). After RA treatment, both hMSC types upregulated the epithelial genes, which anyway reached the highest levels of expression in RA-treated lung-hMSCs (Figure 4B). Of the mesenchymal genes analyzed, both vimentin and snai1 transcripts were more highly expressed at basal level in untreated BM-hMSCs rather than in lung-hMSCs. Following RA treatment, vimentin expression was significantly down-regulated in both BM-hMSCs and lung-hMSCs (Figure 4B), but snai1 expression declined only in lung-hMSCs (Figure 4B).

The expression of vimentin and cytokeratin 18 proteins was analyzed by western blotting on protein extracts to confirm the differences demonstrated at transcriptional level (Figure 4C). Lung-hMSCs always displayed lower expression of vimentin protein before and after RA treatment in comparison to BM-hMSCs (Figure 4C). The expression of the bronchial epithelial protein cytokeratin 18 was detectable only in lung-hMSCs after RA treatment (Figure 4C).

Further characterization of induced epithelial differentiation of lung-hMSCs was carried out by immunofluorescence. This method showed that a small percentage of lung-hMSCs after RA treatment displayed signs of epithelial differentiation, as confirmed by the double-positive staining for cytokeratin 18 and occludin (Figure 4D), the reduction of SMA protein and the expression of E-cadherin (Ecad) (Figure 4D).

At this point it was important to assess whether these molecular changes were associated to the acquisition of specific functional properties, typical of epithelial cells. To this aim, lung-hMSCs were analyzed by Trans Epithelial Electric Resistance (TEER) assay after RA treatment to assess and quantify the formation of tight and adherence junctions among vital cells. As shown in Figure 4E, after RA treatment both BM-hMSCs and lung-hMSCs acquired partial epithelial polarization, typical of “leaky epithelia” (TEER<200 Ω×cm^2^). Full epithelial polarization, specific for “tight epithelia” (TEER>1.000 Ω×cm^2^), was never observed. However, lung-hMSCs resulted more prone to polarization (mean TEER = 60 Ω ×cm^2^) if compared to BM-hMSCs (mean TEER = 40 Ω×cm^2^).

## Discussion

In this study, a population of lung-hMSCs were identified, isolated, expanded and characterized as far as morphology, growth kinetics, immunophenotype, stemness-related gene expression, differentiation potential and immune regulatory properties towards T, B and NK cells are concerned. Altogether, our data show close similarities between lung-hMSCs and BM-MSCs, but also some tissue-specific peculiarities in terms of expression of Nestin and epithelial markers (cytokeratin 18, occludin and tight-junction protein), which confirm the distinct nature of these cells from BM-hMSCs [33], [34]. Lung-hMSC could be identified *in vivo* at perivascular level in the bronchioalveolar region of the lung, as cells co-expressing NG2, CD146 and Nestin and negative for endothelial markers, such as CD31, and CD117 (c-kit). The role of Nestin in lung-hMSCs is unknown. Nestin is an intermediate filament protein that was originally suggested as marker for embryonic and adult stem/progenitor cells of the central nervous system [35]; however, it has been described also in non-neural stem cell populations [36]. Our data are in agreement with previous reports showing the presence of Nestin-positive stem cells with multilineage differentiation capability and mesenchymal phenotype in some epithelial microenvironments, i.e pancreatic gland epithelium [37]. Thus, lung-hMSCs seem to be different from the epithelial stem cells recently described in the lung (c-kit-positive) [38]; lung-hMSCs are more closely related to the MSC progenitors widely diffuse in multiple human organs [11] and obtainable from fetal lung tissues [13] and bronchioalveolar lavage (BAL) from lung-transplanted recipients [14]. Similarly to BM-hMSCs, lung-hMSCs are capable of supporting B cell growth and down-regulating T and NK cell proliferation. The inhibition of T cell proliferation mainly depends on IDO activity; other pathways resulted only partially (heme-oxigenase 1 and IFN-**γ**) or not involved (COX-2/PGE2 and iNOS) in the regulatory effect of lung-hMSCs. PGE2 has been recently suggested as the main soluble factor released by lung-resident MSCs affecting T cell activity [39]. However, the cells described in that paper were not obtained from normal lung tissues, but from BAL of immunocompromized lung-transplanted patients; in addition, the effect of IDO on T cell proliferation was not assessed; thus, the inhibitory machinery of those lung-derived MSCs could have been influenced by the pathological microenviroment [39].

It is known from literature that tissue origin of MSCs may drive their differentiation properties [12]; however, the comparison of hMSCs of different origin, as far as epithelial differentiation is concerned, is still lacking. Our data show that lung-hMSCs after RA treatment can undergo a MET-like process more easily than BM-hMSCs, as shown by the significant up-regulation of three important epithelial genes (cytokeratin 18, occludin and tight-junction protein), together with the down-regulation of the mesenchymal genes vimentin and snai1 (the e-cadherin repressor), whose expression was significantly lower in lung-hMSCs even at basal level. Epithelial commitment was detectable both at protein level, as assessed by Western Blotting and immunofluorescence assays, and at functional level, as confirmed by TEER assay; the latter showed the ability of lung-hMSCs to acquire higher degree of polarization than BM-hMSCs during epithelial differentiation *in vitro*. Thus, lung-hMSCs seem to be constitutively more prone to epithelial differentiation than BM-hMSCs. Accordingly, the higher expression of Nestin transcript and protein by lung-hMSCs could reflect their greater plasticity towards different polarizing signals. Nevertheless, epithelial differentiation reached *in vitro* by lung-hMSCs was partial, as shown by the abnormal localization of the Ecad (typical of the transition state), the weak functional polarization (TEER<200 Ω×cm^2^), and the slight increase of specific lung epithelial transcripts, such as cystic fibrosis trans-membrane conductance regulator protein (cftr) and surfactant proteins (data not shown). These results may reflect either the heterogeneity of MSC population or the inadequacy of the stimuli used for MET induction. However, RA was the strongest *in vitro* epithelial differentiation-inducing stimulus in our hands, as the addition of other factors, such as EGF, IGF, HGF or FGF10 did not significantly change the effects obtained with RA alone (data not shown). We also tried to induce epithelial differentiation of hMSCs by using the supernatant obtained from immortalized lung epithelial cell lines (16hbe or A549); however, this approach led only to the upregulation of some specific epithelial markers rather than to organized MET (data not shown). These results are in agreement with several papers pointing out the essential role of RA in lung development [16]–[21], [23], [25], and as molecular inducer of cell differentiation [26]–[28] and postnatal recovery factor [22], [29], [30]. Nevertheless, the *in vivo* contribution of retinoids to the activation and induction of the epithelial differentiation of lung-hMSCs needs to be preliminarily assessed in *in vivo* models of lung regeneration following tissue damage, before speculating about any possible usefulness of lung-hMSCs in pulmonary regenerative medicine.

By contrast, lung-hMSC characterization here described may be useful to study a variety of lung diseases, such as chronic obstructive pulmonary disease (COPD) and idiopathic pulmonary fibrosis (IPF). In fact, both diseases are characterized by abnormal remodelling of alveolar spaces (emphysema and bronchiolitis in COPD, irreversible fibrosis in IPF), which is probably associated to the severe impairment of the cross-talk between the two pulmonary stem cell compartments (alveolar epithelial precursors and mesenchymal progenitors), together with a poorly characterized dysregulation of the local immunity [40]–[45]. Alveolar epithelial stem cells (type II pneumocytes) are mainly affected in IPF [46]–[48], whereas most damages affect mesenchymal precursor cells within the alveolar parenchyma in COPD [49]–[51]. Abnormal senescence of mesenchymal precursors in emphysema leads to parenchyma loss, defective production of extracellular matrix proteins, such as elastin and fibronectin, and progressive weakening of the alveolar structures [49]–[55]. In addition, it is likely that the immune regulatory properties of lung mesenchymal precursors are affected in COPD pathogenesis, as abnormal immunological and inflammatory reactions have been described in this disease [40], [55]–[57]. Thus, the availability of normal lung-hMSCs may represent a good model *in vitro* to assess at immunophenotypic, molecular, and functional levels the pathological changes occurring in COPD and IPF, and to study the reciprocal interactions between epithelial stem cells and MSCs of pulmonary origin in normal and pathological conditions.
